# The effects of urbanization, temperature, and rainfall on *Aedes aegypti* and *Aedes albopictus* mosquito abundance across a broad latitudinal gradient in Central Africa

**DOI:** 10.1186/s13071-025-06764-5

**Published:** 2025-04-06

**Authors:** Matthew J. Montgomery, James F. Harwood, Aurelie P. Yougang, Théodel A. Wilson-Bahun, Armel N. Tedjou, Christophe Rostand Keumeni, Charles S. Wondji, Basile Kamgang, A. Marm Kilpatrick

**Affiliations:** 1 U.S. Naval Medical Research Unit EURAFCENT, PSC 824, Box 23, FPO AE 09623 Naval Air Station Sigonella, Italy; 2 Ecology and Evolutionary Biology Department, University of California Santa Cruz, 130 McAllister Way, Santa Cruz, CA 95060 USA; 3 Centre for Research in Infectious Diseases, P.O. Box 13591, Yaoundé, Cameroon

**Keywords:** Mosquito, Urbanization, Arbovirus, Africa, Vector-borne disease, Climate change

## Abstract

**Background:**

Urbanization can influence disease vectors by altering larval habitat, microclimates, and host abundance. The global increase in urbanization, especially in Africa, is likely to alter vector abundance and pathogen transmission. We investigated the effect of urbanization and weather on the abundance of two mosquitoes, *Aedes aegypti* and *Aedes albopictus*, and infection with dengue, chikungunya, and Zika viruses at 63 sites in six cities spanning a 900-km latitudinal range in Cameroon, Central Africa.

**Methods:**

We used human landing catches and backpack-mounted aspirators to sample mosquitoes and collected larval habitat, host availability, and weather (temperature, precipitation, humidity) data for each site in each city. We analyzed land use and land cover information and satellite photos at varying radii around sites (100 m to 2 km) to quantify the extent of urbanization and the number of structures around each site. We used a continuous urbanization index (UI; range 0–100) that increased with impermeable surface and decreased with forest cover.

**Results:**

Urbanization increased larval habitat, human host availability, and *Ae. aegypti* mosquito abundance. *Aedes aegypti* abundance increased 1.7% (95% CI 0.69–2.7%) with each 1 unit increase in the urbanization index in all six cities (Douala, Kribi, Yaounde, Ngaoundere, Garoua, and Maroua) with a 5.4-fold increase from UI = 0 to UI = 100, and also increased with rainfall. In contrast, *Ae. albopictus* abundance increased with urbanization in one city, but showed no influence of urbanization in two other cites. Across three cities, *Ae. albopictus* abundance increased with rainfall, temperature, and humidity. Finally, we did not detect Zika, dengue, or chikungunya viruses in any specimens, and found weak evidence of interspecific competition in analyses of adult population growth rates.

**Conclusions:**

These results show that urbanization consistently increases *Ae. aegypti* abundance across a broad range of habitats in Central Africa, while effects on *Ae. albopictus* were more variable and the abundance of both species were influenced by rainfall. Future urbanization of Africa will likely increase *Ae. aegypti* abundance, and climate change will likely alter abundance of both species through changes in precipitation and temperature.

**Graphical Abstract:**

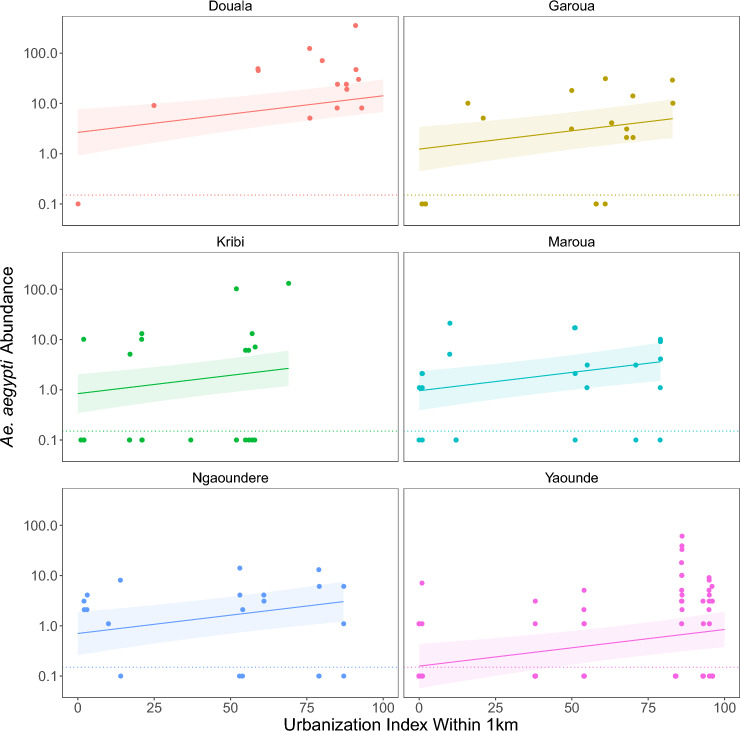

**Supplementary Information:**

The online version contains supplementary material available at 10.1186/s13071-025-06764-5.

## Background

Urbanization, the conversion of natural and agricultural habitats to urban areas, alters the biotic and abiotic conditions faced by organisms and is increasing globally [1, 2]. Urbanization often reshapes community composition, with introduced and human commensal species replacing native species [3]. Urbanization can also alter microclimates, including increasing temperature through the heat island effect and reducing humidity by reducing vegetation [4]. These changes in the biotic and abiotic environment can also influence the transmission of pathogens [5], including those that cause vector-borne disease [6]. The effect of urbanization on vector-borne disease depends on both the response of hosts and vectors to urbanization and the effects of microclimate variation on pathogen development in the vector [7]. All these factors can increase or decrease with urbanization depending on the vectors, hosts, pathogen, and local climate [8–11]. Africa is a key area for understanding the effect of urbanization on vector borne disease because it has the world’s highest rates of urban growth [12] and many vector-borne diseases. *Aedes*-borne arboviruses, including dengue, chikungunya, and Zika viruses, are an especially important group of pathogens because of their broad geographic distribution, disease burden, and recent increases in incidence and outbreaks [13, 14].

Urbanization can increase larval habitat, especially for key anthropogenic species such as *Aedes aegypti* and *Aedes albopictus*, through increased abundance of refuse, containers, and other objects that retain water [15–18]. Furthermore, urbanization is often associated with water storage for human use throughout the year, which can provide mosquito larval habitat in periods with low rainfall, especially in arid climates [16]. Temperature increases associated with urbanization can alter mosquito community composition and pathogen transmission through multiple mechanisms. Temperature tolerance (both minimum and maximum, as well as daily variation) varies considerably between mosquito species. Temperature influences survival, biting rate, development rate, fecundity, and vector competence [19–23], as well as the extrinsic incubation period of many pathogens [19]. Increasing temperatures with urbanization can decrease larval development time, the extrinsic incubation period, and adult survival, which can have opposing effects on transmission [10, 24–26]. In contrast, decreased humidity generally reduces mosquito survival without any positive effects on transmission [27]. Urbanization can also alter interspecific competition among mosquito larvae; For example, larval habitat that entirely dries out favors *Ae. aegypti* over *Ae. albopictus*, whereas the inverse is true in larval habitat that never entirely dries out [28].

The net effects of urbanization on mosquito abundance can vary among species. *Ae. aegypti* abundance increases with urbanization across its global range, with very few exceptions (Supplementary Table S1). In contrast, the effects of urbanization on *Ae. albopictus* abundance appear to be more variable, with increases in 8 of 15 previous studies, decreases in 6, and no effect in 1 (Supplementary Table S2); For example, *Ae. albopictus* abundance was higher in urban than rural areas in China [17] and Mayotte [29], but abundance was highest in suburban/transitional areas in Virginia, USA [30] and Thailand [31] and rural areas in Brazil [32] and Florida, USA [33]. A key area where the effect of urbanization on *Ae. albopictus* needs additional study is the continent of Africa [34]. In Central Africa, only very limited data were used to examine factors influencing the presence or absence of this species, and in most cases no data on mosquito abundance were analyzed [34–36]. While *Ae. aegypti* is native to central Africa, *Ae. albopictus* is an introduced species that was first detected in 2000 [37].

Our goal was to examine the effect of urbanization on the abundance and arbovirus infection rates of *Ae. aegypti* and *Ae. albopictus* mosquitoes across a broad latitudinal gradient in Central Africa. We examined adult mosquito abundance, weather, microclimates, larval habitat, and host abundance at ten sites in each of six cities spanning the 900-km latitudinal extent of Cameroon in Central Africa. We also tested *Aedes* mosquitoes for dengue, chikungunya, and Zika viruses, which are important arboviruses that circulate in this region [38]. We studied adult mosquitoes rather than mosquito larvae, which have been the focus of some earlier work [13, 21, 23, 24], so we could detect pathogens and because adult mosquito abundance is often a better predictor for human outbreaks than larval indices [39]. We hypothesized that *Ae. aegypti* would increase with urbanization, but we predicted that the slope would vary among cities because the benefits of urbanization would be lower in areas that were hotter and dryer. We hypothesized that *Ae. aegypti* abundance would also be influenced by *Ae. albopictus* abundance, which might show increasing or decreasing relationships with urbanization. In some regions of the world *Ae. albopictus* competes with and can displace *Ae. aegypti* populations through larval competition and reproductive interference [40]. We examined multiple scales of urbanization (100 m to 2 km) on mosquito abundance and hypothesized that small-scale measures of urbanization (100 m to 200 m) would most closely correlate with abundance, given the short-distance dispersal distances of *Ae. aegypti* and *Ae. albopictus* in mark–recapture studies [41, 42].

## Methods

### Study area

**Fig. 1:**
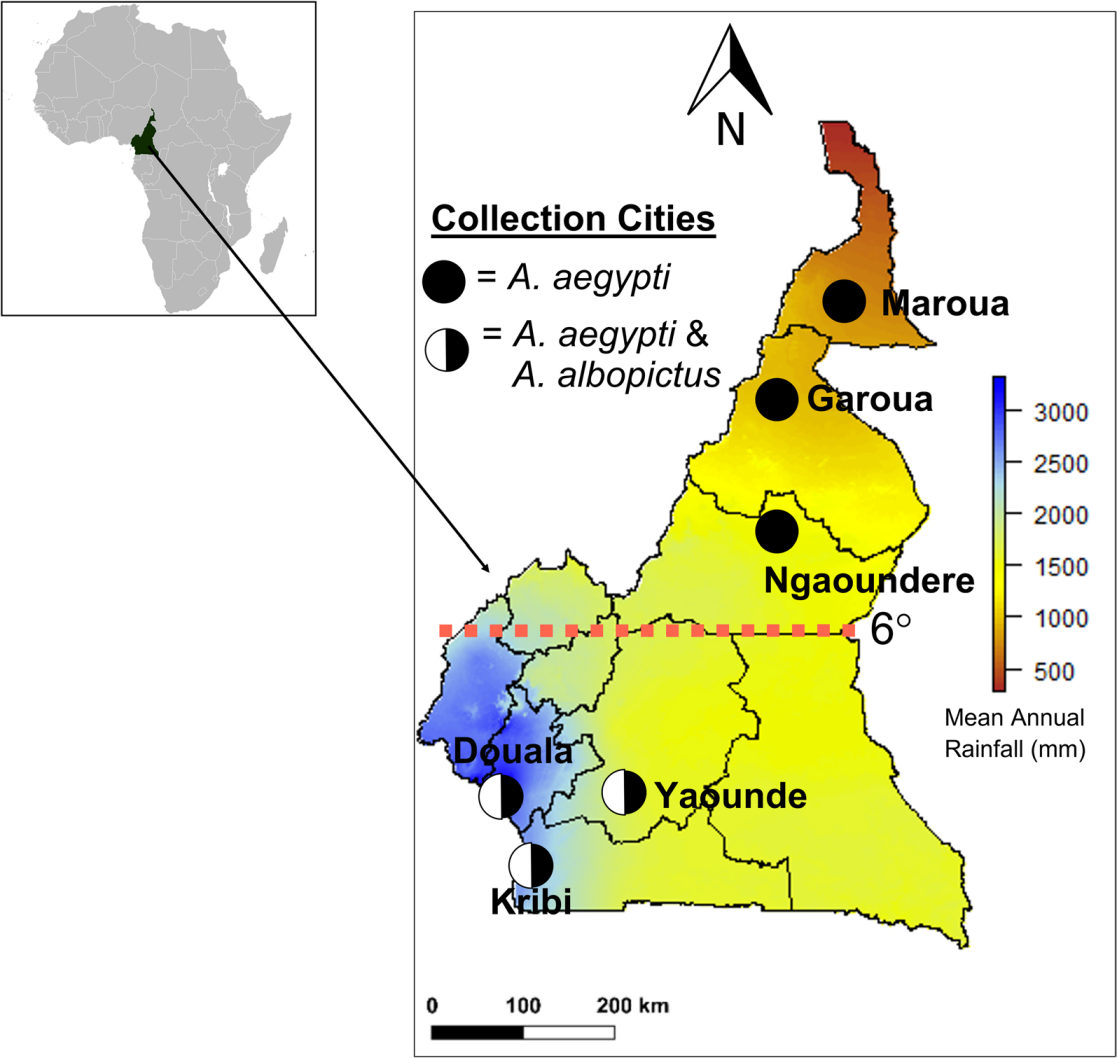
The six cities we surveyed for adult mosquitoes in Cameroon, Central Africa, spanned a large rainfall gradient. Above approximately 6° latitude, *Ae. albopictus* was not found

We collected mosquitoes in six cities spanning a 900-km latitudinal gradient in Cameroon (Fig. 1) representing five African ecoregions: Atlantic equatorial coastal forest (Douala, population 3.8 Million; Kribi, population ~60,000), Congolian lowland forest (Yaounde, population 2.8 M), Northern Congolian Forest Savannah mosaic (Ngaoundere, population 1.2 M), East Sudanese Savannah (Garoua, population 2 M), and Sahel (Maroua, population 1.4 M) [43]. Average annual temperatures and relative humidity varied across all cities between 18 and 28 °C and 45% to 85%. Cities ranged in altitude from 2 to 1200 m [44] and spanned a large precipitation gradient (Fig. 1). We sampled at approximately ten sites across an urbanization gradient in each of the six cities, from forest to agrarian areas to dense urban centers. The sampling was both cross-sectional, with each site in five cities being sampled only twice, and also longitudinal, with sites in Yaounde being sampled 9–21 times.

### Land use analysis

We quantified the urbanization around each site at 100 m, 200 m, 500 m, 1 km, and 2 km radii using an urbanization index (UI) [45], where

$\text{UI}=\frac{(100\% - \% vegetation cover+\% impervious surface)}{2}$. An intact forest would have an UI of 0, whereas a fully impermeable surface (e.g., paved roads or buildings) would have a value of 100. We performed spatial analyses using ArcGIS Pro version 2.8.7. We imported GPS field coordinates into ArcGIS Pro and obtained spatial landcover data from the European Space Agency’s (ESA) Climate Change Initiative—S2 Prototype Land Cover 20 M map of Africa 2016 [46]. We also used satellite imagery (Google Earth Pro version 7.3.4.8642) to visually quantify the number of structures at the 100 m radius for each surveillance site.

### Urbanization and mosquito larval habitat

To investigate how urbanization altered the available of mosquito larval habitat, we searched for potential larval habitat (all containers that could hold water) within 50 m of each collection site in all cities except Yaounde. We categorized and sampled any container with mosquito larvae present, and any suspected *Ae. aegypti* and *Ae. albopictus* larvae collected were reared to adulthood and identified to species.

### Adult mosquito collection

We collected adult mosquitoes outdoors using two stationary human collectors using Modified Centers for Disease Control Backpack Aspirators (John W. Hock Company, Gainesville, Florida). We used human landing catches, rather than passive traps, to increase the number of adult *Aedes* captured and the chance of detecting and accurately measuring arbovirus infection rates [47]. We conducted collections for 3 h during one of two 3-h time periods (8–11 am and 3 pm to 6 pm) corresponding to *Ae. aegypti* and *Ae. albopictus* activity peaks in Africa [48]. We recorded temperature and humidity at the start of each collection period, and obtained data on rainfall for our study cities [49]. We performed the 3-h collections one or two times at each of the ~10 sites in each city over a 1-week period. We sampled each city (except Yaounde) once during the dry and once during rainy seasons from between September 2020 and August 2021. In Yaounde, we visited each site approximately ten times between September 2020 and October 2021.

We killed adult mosquitoes by freezing them at −20 °C or using a chloroform solution and identified mosquitoes using morphological identification keys [50]. We grouped *Aedes* females by species and pooled them in groups of 3–30 individuals (mean 23.4) and stored them at −80 °C in RNA*later*, following the manufacturer’s instructions for viral detection (LifeTechnologies, 2011).

### Pathogen detection

We ground pools of 3–30 mosquitoes in 200 µL of Leibovitz L15 medium equilibrated at room temperature and then centrifuged samples for 15 min at 15,000 rpm before transferring the supernatant to a new Eppendorf tube. We extracted total viral RNA using the Qiagen extraction kit (QIAamp-viral RNA mini kit, Qiagen) following the manufacturer’s instructions and stored samples at −80 °C before testing samples by quantitative polymerase chain reaction (qPCR) for dengue, Zika, and chikungunya viruses (see details in Supplementary Material).

### Urbanization and host availability

To investigate how urbanization altered the available of mosquito hosts, in Douala, Kribi, and Yaounde, we counted potential hosts using two stationary observers from 8 to 11 am and from 3 pm to 6 pm on two separate days at each established surveillance site. We recorded all humans, chickens, goats, cows, and dogs visible over a 5-min period. We counted human hosts to a maximum of 100 individuals owing to extremely large crowds in some sites rendering more accurate counts unfeasible.

### Indoor versus outdoor mosquito abundance

We also investigated whether our outdoor mosquito abundance along the urbanization gradient correlated with indoor household mosquito abundance by conducting paired surveillance inside ten homes of willing households within 50 m of the outdoor collection site at each of ten sites in Yaounde in October of 2021 using the same Modified Centers for Disease Control Backpack Aspirators.

### Competition between *Ae. aegypti *and* Ae. Albopictus*

We calculated monthly population growth rates (*λ* = *N*_*t*+1_/*N*_*t*_) for each species for the two sites in Yaounde from which adequate numbers of both species were simultaneously collected. We then examined the potential effects of interspecific competition between *Ae. aegypti* and *Ae. albopictus* by examining the effect of the abundance of *Ae. albopictus* on the log_10_ population growth rate *λ* over the following month for *Ae. aegypti*, and vice versa using linear regression. We included the abundance of the focal species in these analyses to account for intraspecific competition.

### Statistical analyses

Our primary goal was to determine how urbanization and weather (temperature and precipitation) influenced abundance for both mosquito species. As a part of this goal, we determined which spatial scale (radius around each site) of urbanization best predicted mosquito abundance. Next we wanted to determine whether larval habitat or host abundance was responsible for the correlations we found between the urbanization index and mosquito abundance. To accomplish these goals, we analyzed mosquito abundance using generalized linear mixed effect models with a negative binomial distribution and log link using the lme4 package in R version 3.6.1. We included city as a fixed effect, site as a random effect, and the number of hours of sampling as an offset. We used Akaike’s information criterion (AIC) to compare models with different urbanization index radii, while including the effects of temperature, humidity, and rainfall. We also used AIC to compare interactive versus additive models for the effects of urbanization among cities, while including temperature, humidity, and rainfall.

After determining the best fitting urbanization index radius and additive or interactive model of city and urbanization, we then used to AIC to determine whether the sampling month’s rainfall was better than two other rainfall metrics considered individually (prior month’s rainfall, and the no. of days in the sampling month with rain), while including the other predictors (urbanization index, temperature, humidity). To determine whether larval habitat or human hosts was the mechanism underlying correlations between mosquito abundance and urbanization, we first correlated the number of larval habitat containers and number of human hosts with the urbanization index in separate analyses, both with a negative binomial distribution and log link and site as a random effect. We then used AIC to compare models with the urbanization index to identical models where we replaced the urbanization index with one of the larval habitat indices (containers, larval positive containers, fraction of containers with larvae (container index), no. of larvae, or no. of structures) or our estimates of human host density. The results of each statistical model is given in the Supplementary Material.

## Results

### Larval habitat survey

**Fig. 2:**
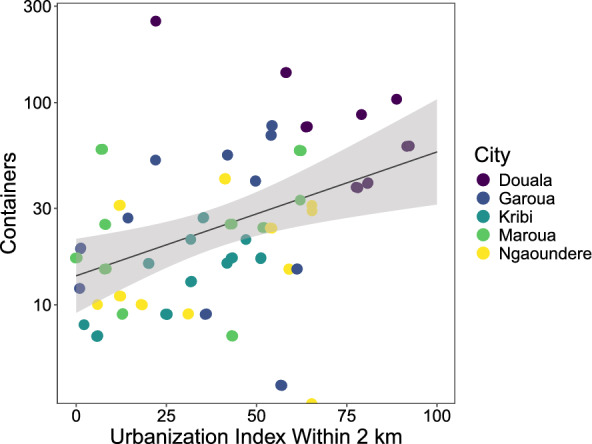
Number of larval habitat containers plotted against the urbanization index within 2 km for five of the study cities. Line and ribbon show the fitted model and 95% CI, respectively

Larval habitat containers increased 4.1-fold across the full urbanization gradient (UI = 0 to UI = 100), with 2 km and 500 m being the best fitting radii (Supplementary Table S4; Ln(containers) = 2.63 + 0.014 (SE = 0.0046) × UI_2KM; P = 0.0023; site random effect variance: 0.63; Fig. 2). Larvae-positive containers increased 6.6-fold with the highest rates of urbanization, and 2 km was the best fitting radius (Supplementary Table S5; Ln(positive containers) = 1.72 + 0.019 (SE = 0.0053) × UI_2KM; P = 0.00035; site random effect variance: 0.80). There was no significant relationship between the container index (larvae occupied containers/available containers) and urbanization at any radius (all *P* > 0.29).

### Host availability

Human abundance increased significantly with UI (increasing 8.8-fold from 6.1 to 53.9 as UI increased from 0 to 100; Additional File 1: Supplementary Fig. S1), and 1 km was the best fitting radius (Table S6; Ln(humans) = 1.81 + 0.022 (SE = 0.0064) × UI_1KM; *P* = 0.00062; site random effect variance: 0.96), while animal abundance decreased 26-fold with UI from 4.8 to 0.18 as UI increased from 0 to 100, and 200 m was the best correlate (Supplementary Table S7; Ln(humans) = 1.57 − 0.033 (SE = 0.0066) × UI_200M; *P* = 7.6 × 10^–7^, site random effect variance: 0.44).

### Heat island effect

Neither temperature nor humidity was significantly correlated with the urbanization index at any radii, in models with city and site as a random effect (temperature: all *P* > 0.64; humidity: all *P* > 0.36).

### Mosquito abundance

We collected 25,853 male and female adult mosquitoes from 231 two-person collection sessions from 63 sites across 6 cities. Of these mosquitoes, 15,008 were *Ae. albopictus* (9,053 females) and 2633 were *Ae. aegypti* (1,691 females), and the remainder were primarily other *Aedes*, or *Culex*, *Culiseta*, or *Anopheles* mosquitoes. No *Ae. albopictus* were found in our three study cities north of 6° latitude (Ngaoundere, Garoua, and Maroua).

**Fig. 3:**
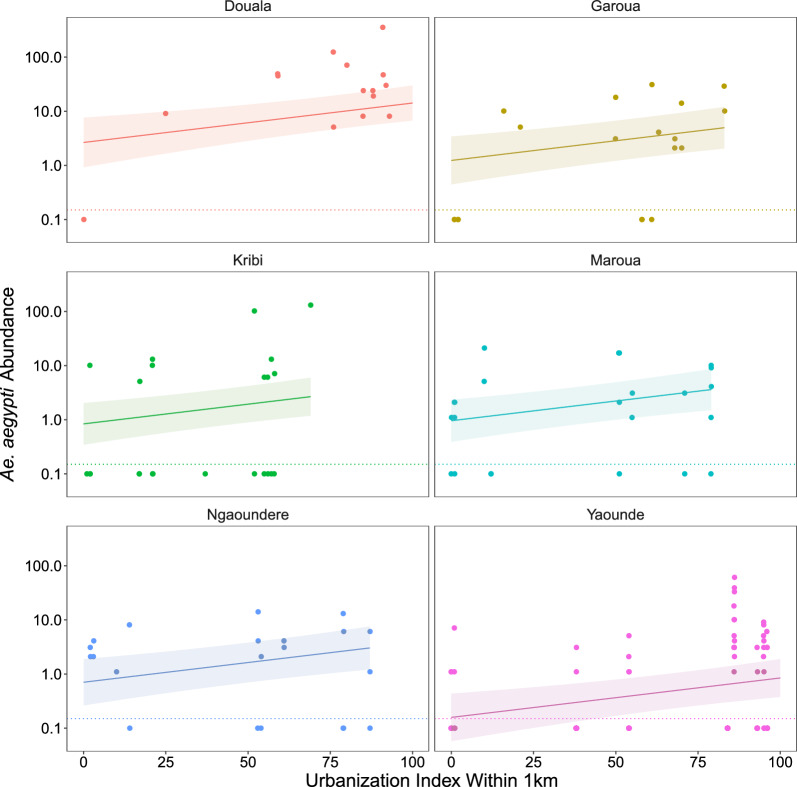
*Ae. aegypti* abundance (mosquitoes/3 h) on a log_10_ scale plotted against the urbanization index in a 1-km radius around study sites for ten sites in each of six cities. Collections resulting in zero specimens (below the dotted lines) were plotted at 0.1. Lines and ribbons show fitted models with UI at 1 km as a predictor and 95% CI, respectively (Supplementary Table S8)

**Fig. 4:**
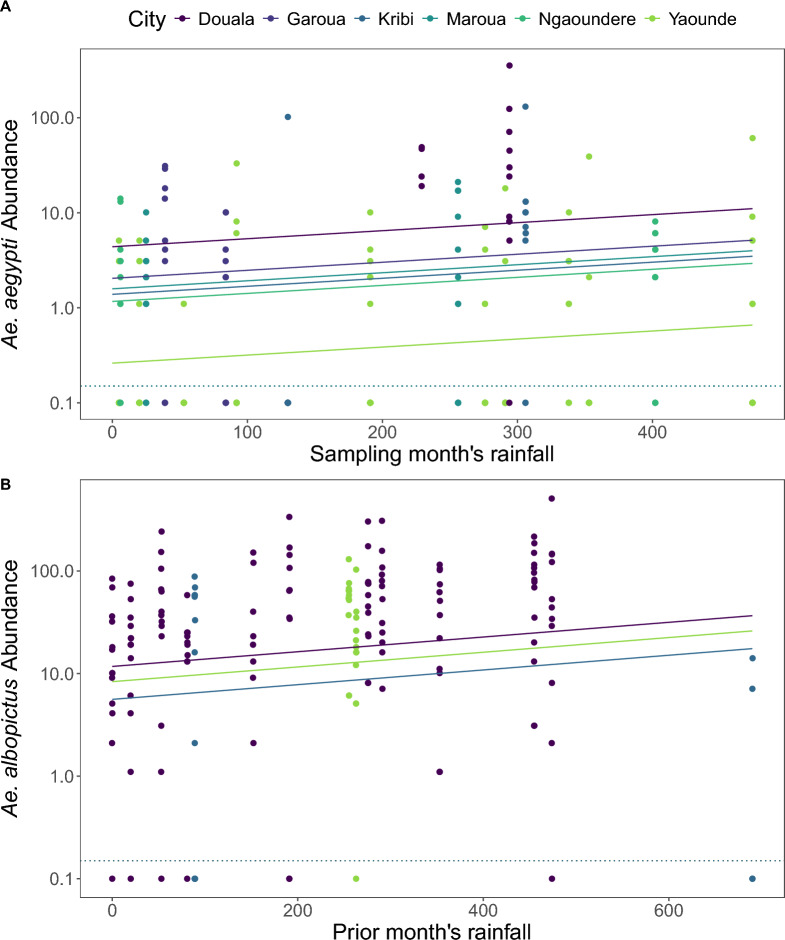
*Aedes aegypti* (**A**) and *Ae. albopictus* (**B**) abundance (mosquitoes/3 h) on a log10 scale plotted against the sampling month’s (**A**) or prior month’s (**B**) rainfall. Collections resulting in zero specimens (below the dotted lines) were plotted by adding 0.1. Lines show fitted models.

*Ae. aegypti* abundance increased with UI in all cities (Fig. 3), and 1 km was the best fitting radius (Supplementary Table S8). *Aedes aegypti* abundance also increased with rainfall (Fig. 4A) but was uncorrelated with humidity and temperature (Supplementary Table S9), despite both varying substantially among and within sites over time (Additional file 1: Supplementary Fig. S2). The slope of the response to urbanization was consistent across cities (an additive model fit better than a model with a city × UI interaction; ∆AIC 2.57), with a 1.7% (95% CI 0.69–2.7%) increase (e^(0.017)^ = 1.017) in *Ae. aegypti* abundance for each 1 unit increase in UI (*i.e.*, a 1.017^100^ = 5.4-fold increase from UI = 0 to UI = 100). *Aedes aegypti* abundance varied considerably between cities, with the greatest mean abundance in Douala and the lowest in Yaounde (Fig. 3; Supplementary Table S9). In the City of Yaounde, where we sampled almost monthly, *Ae. aegypti* abundance peaked during the month of highest rainfall (Additional file 1: Supplementary Fig. S3). Across all cites, the sampling month’s rainfall was a better predictor than the previous month (ΔAIC 5.62) or the number of days in the sampling month with rain (ΔAIC 4.59).

In cities where larval surveys were done (all but Yaounde), there was no correlation between *Ae. aegypti* abundance and the number of containers, larvae-positive containers, or container index (all *P* > 0.43), but abundance increased marginally with the number of structures in a 100-m radius (Supplementary Table S10). *Aedes aegypti* abundance increased significantly with human abundance in the three cities where human surveys were performed (2.2% for each human; Supplementary Table S11), and the correlation with humans was even stronger than with the UI (ΔAIC 2.05).

**Fig. 5:**
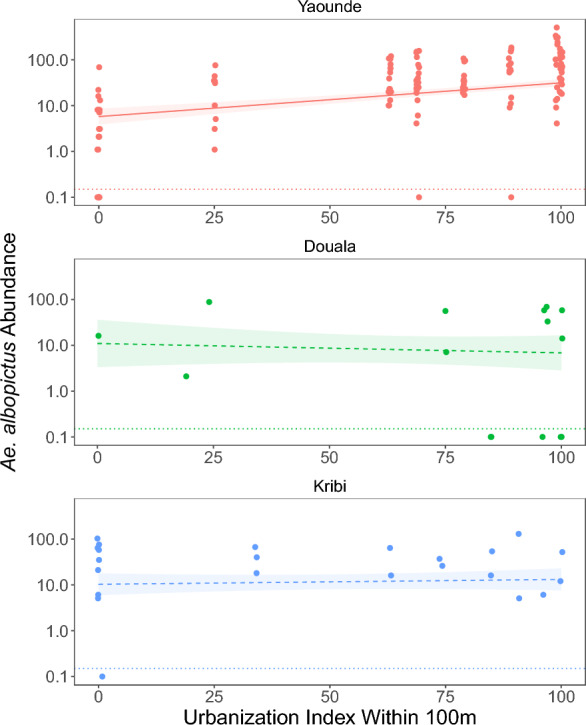
*Aedes albopictus* abundance (mosquitoes/3 h) on a log_10_ scale plotted against the urbanization index in a 100 m radius around study sites for ten sites in each of three cities where *Ae. albopictus* was found. Lines and ribbons show fitted model and 95% CI, respectively (Supplementary Table S6). The slope of urbanization in Douala and Kribi was not significantly different from zero. Collections resulting in zero specimens (below the dotted lines) were plotted at 0.1

*Aedes albopictus* showed heterogeneity in its response to urbanization in the three cities where it was found (Fig. 5), and the best fitting UI radius was 100 m (Supplementary Table S12). In Yaounde, *Ae. albopictus* increased significantly with urbanization, but there was no significant pattern in either Kribi or Douala (Supplementary Table S13; Fig. 5). *Aedes albopictus* abundance also increased with temperature, humidity, and the previous month’s rainfall (Fig. 4B), which was a better predictor than the sampling month’s rainfall (ΔAIC 14.7) or the sampling month’s days with rainfall (ΔAIC 7.2) (Supplementary Table S13).

In the two cities with *Ae. albopictus* where larval habitat surveys were done (Kribi and Douala), adult mosquito abundance increased with the number of larval habitat containers (Supplementary Table S14), containers with larvae (Supplementary Table S15), and number of *Ae. albopictus* larvae (Supplementary Table S16), but decreased with the fraction of all containers containing larvae (Supplementary Table S17), and decreased, weakly, with the number of structures in a 100-m radius (Supplementary Table S18). *Aedes albopictus* abundance increased with human density (Supplementary Table S19), but the correlation was not as strong as with UI (ΔAIC 18.5).

### Pathogen detection

We tested 1660 Ae. aegypti and 7771 *Ae. albopictus* females for chikungunya, dengue, and Zika viruses in 402 pools of *N*_avg_ = 23.4 mosq./pool (*N*_avg_ = 67 pools/city; range 6–247; _avg_ = 1569 mosq/city, range 36–6067). No viruses were detected.

### Indoor/outdoor abundance

Across all ten surveillance sites in Yaounde, no *Ae. aegypti* or *Ae. albopictus* mosquitoes were caught during indoor collections, while simultaneous outdoor collections caught a total of 28 and 397 mosquitoes, respectively.

### Competition between species

There was no detectable effect of adult *Ae. albopictus* on adult *Ae. aegypti* population growth rates (Additional file 1: Supplementary Fig. S4A: Log(*Ae. aegypti* pop. growth rate) = 0.082  − 0.0089 × *Ae. aegypti* (SE = 0.0087) + 0.00042 × *Ae. albopictus* (SE = 0.0037); *P* = 0.33 and *P* = 0.91 for intra- and interspecific competition respectively), and little evidence of intraspecific density dependence, differences among season, or variation among sites (all *P* > 0.47). However, adult *Ae. albopictus* population growth rates showed clear evidence of intraspecific density dependence and weak evidence of competition from adult *Ae. aegypti* (Additional file 1: Supplementary Fig. S4B; Log(*Ae. albopictus* pop. growth rate) = 0.67 − 0.0083 × *Ae. albopictus* (SE = 0.0017) − 0.0074 × *Ae. aegypti* (SE = 0.0041); *P* = 0.00046 and *P* = 0.095, respectively). As with *Ae. aegypti*, there was little variation in the *Ae. albopictus* population growth rate among seasons or sites (*P* > 0.27).

## Discussion

We found that *Ae. aegypti* abundance increased consistently with urbanization across the latitudinal extent of Cameroon, despite substantial climatic differences along this 900-km north–south gradient. We had predicted that the increase in *Ae. aegypti* abundance with urbanization would be smaller in the hottest, most arid environments, but we found no evidence that hotter climates limited the positive influence of urbanization, possibly because we found no relationship between urbanization and temperature within cities. In contrast, *Ae. albopictus* increased with urbanization in one city but showed no significant relationship with urbanization in two other cities. This variable response in Central Africa mirrors the variable response of this species globally (Additional file 1: Supplementary Fig. S5).

We investigated several mechanisms by which urbanization could influence mosquito abundance, including larval habitat availability, host abundance, and microclimates. Larval habitat increased with urbanization, but the urbanization index was more strongly correlated with *Ae. aegypti* abundance than all larval habitat predictors, at the sites where larval habitat studies were performed. Human abundance also increased with urbanization, and *Ae. aegypti* abundance was even more highly correlated with human abundance than the urbanization index at the three cities where human abundance was measured. In contrast, *Ae. albopictus* abundance was strongly correlated with most measures of larval habitat in the two cities with this species where larval habitat studies were performed. *Aedes albopictus* abundance also increased with human abundance, but not as strongly as with the urbanization index.

The spatial scale of urbanization that best correlated with mosquito abundance was relatively large for *Ae. aegypti* (1 km) and varied between species (100 m for *Ae. albopictus*). The large radius for *Ae. aegypti* ran counter to our expectation that the more local scale measure (100 m) would correlate more strongly with abundance. These results indicate that urbanization affects populations on a scale larger than the typical dispersal distance of an individual mosquito. Human host availability, which was strongly correlated with *Ae. aegypti* abundance, was most correlated with urbanization at 1 km, which might help explain why this species also responded to urbanization at larger spatial scales.

We detected no chikungunya, dengue, or Zika viruses in either mosquito species, despite testing more mosquitoes (over 9000) than other previous studies that did detect dengue and chikungunya virus in *Ae. aegypti* and *Ae. albopictus* (Supplementary Table S20; 280–5000). The low infection prevalence generally observed for these pathogens in mosquitoes makes studies of human disease risk difficult [51]. Our large sample size and no virus detection for chikungunya, dengue, or Zika suggests that these viruses were circulating at low rates during the times and at the places we surveyed. Nonetheless, these viruses definitely circulate locally: there were 41 documented human dengue cases in one of our cities, Douala, during our study [52], and a recent (2015) nationwide serological survey of 1084 Cameroonian blood donors found a 5% seroprevalence for Zika virus overall and 10% seroprevalence in Douala [53].

In Yaounde, we found no *Ae. aegypti* or *Ae. albopictus* during indoor household surveys, while simultaneous outdoor surveys captured numerous mosquitoes, albeit with different trapping methods (human landing catches outside, backpack aspirating indoors). This supports prior research describing both species as exophilic [48]. Vector control measures that reduce human outdoor exposure to these mosquitoes are needed to reduce arboviruses transmitted by these mosquitoes.

## Conclusions

Africa is one of the most rapidly urbanizing regions on Earth [54]. The six cities in this study are broadly representative of the major ecotypes of sub-Saharan Africa, suggesting that *Ae. aegypti* likely increases with urbanization across a wide portion of the continent. The relationship we estimated (a 1.7% increase for every 1 unit increase in UI, or a 1.017^100^ = 5.4-fold increase from UI = 0 (natural land cover with no impermeable surfaces) to UI = 100 (fully impermeable surfaces)) could be used to estimate changes in abundance with increasing urbanization. In contrast, our results indicated that *Ae. albopictus* abundance will likely increase with urbanization in some places but not in others. We found that both species increased with rainfall, highlighting the large importance of this climatic variable. The combination of urbanization and increased precipitation in Central Africa with climate change [55] is likely to increase the abundance of both these species, and possibly the viruses they transmit.

## Supplementary Information

Additional file 1: Text S1. Pathogen detection methods: RNA extraction and RT-PCR. Fig. S1. Humans observed at collection sites in Douala, Kribi, and Yaounde. Fig. S2. Relative humidity (in percent) and temperature (in degrees Celsius) measurements from each of the six cities. Fig. S3. Monthly rainfall and mosquito abundance (mosquitoes/3 h) for ten sites in Yaounde. Fig. S4. Monthly population growth rate of *Ae. aegypti* plotted against abundance of *Ae. albopictus* in the preceding month (A) and vice versa (B). Table S1. Previous studies on *Ae. aegypti* abundance and urbanization. Table S2. Previous studies on *Ae. albopictus* abundance and urbanization. Table S3: Sequence of primers used for viral detection. Table S4. Model comparison of different radii for urbanization index predicting the number of larval habitat containers. Table S5. Model comparison of different radii for urbanization index predicting the number of larval habitat containers with mosquito larvae. Table S6. Model comparison of different radii for urbanization index predicting the number of human hosts. Table S7. Model comparison of different radii for urbanization index predicting the number of animal hosts. Table S8. Model comparison of different radii for urbanization index predicting the abundance of *Ae. aegypti* mosquitoes. Table S9. Analysis of *Ae. aegypti* abundance in six cities with urbanization index (UI), with temperature, humidity, and the sampling month’s rain as predictors. Table S10. Analysis of *Ae. aegypti* abundance in five cities (all but Yaounde) with structures within 100 m, city, temperature, humidity, and sampling month’s rain as predictors. Table S11. Analysis of *Ae. aegypti* abundance with number of humans, temperature, humidity, and monthly rainfall as predictors. Table S12. Model comparison of different radii for urbanization index predicting the abundance of *Ae. albopictus* mosquitoes. Table S13. Analysis of *Ae. albopictus* abundance in the three cities where this species was found, with urbanization index (UI) interacting with city, and temperature, humidity, and prior month’s rain as predictors. Table S14. Analysis of *Ae. albopictus* abundance in the two cities where this species was found, and larval habitat studies were performed (Douala and Kribi), with larval containers, city, temperature, humidity, and prior month’s rain as predictors. Table S15. Analysis of *Ae. albopictus* abundance in the two cities where this species was found, and larval habitat studies were performed (Douala and Kribi), with larval containers with mosquito larvae (pos. containers), city, temperature, humidity, and prior month’s rain as predictors. Table S16. Analysis of *Ae. albopictus* abundance in the two cities where this species was found, and larval habitat studies were performed (Douala and Kribi), with larval containers with *Ae. albopictus* mosquito larvae (Alb. larvae), city, temperature, humidity, and prior month’s rain as predictors. Table S17. Analysis of *Ae. albopictus* abundance in the two cities where this species was found, and larval habitat studies were performed (Douala and Kribi), with the fraction of larval containers containing mosquito larvae (Cont. index), city, temperature, humidity, and prior month’s rain as predictors. Table S18. Analysis of *Ae. albopictus* abundance in the two cities where this species was found, and larval habitat studies were performed (Douala and Kribi), with Structures within 100 m, city, temperature, humidity, and prior month’s rain as predictors. Table S19. Analysis of *Ae. albopictus* abundance in the three cities where this species was found, with humans, city, temperature, humidity, and prior month’s rain as predictors. Table S20. Compiled minimum infection rates (MIR) for studies that detected Zika virus in field-caught specimens of *Ae. aegypti* and *Ae. albopictus*.

## Data Availability

The data and code underlying the results of this manuscript will be provided upon reasonable request to the authors.

## Abbreviations

UI: Urbanization index
AIC: Akaike’s information criterion
CI: Confidence interval
