# Supplementary material for: The effects of urbanization, temperature, and rainfall on Aedes aegypti and Aedes albopictus mosquito abundance across a broad latitudinal gradient in Central Africa
Source: Parasit Vectors. 2025 Apr 6;18:135. doi: 10.1186/s13071-025-06764-5 (PMC11972486; doi:10.1186/s13071-025-06764-5)
Supplement: Supplementary file 1 — Additional file 1: Text S1. Pathogen detection methods: RNA extraction and RT-PCR. Fig. S1. Humans observed at collection sites in Douala, Kribi, and Yaounde. Fig. S2. Relative humidity (in percent) and temperature (in degrees Celsius) measurements from each of the six cities. Fig. S3. Monthly rainfall and mosquito abundance (mosquitoes/3 h) for ten sites in Yaounde. Fig. S4. Monthly population growth rate of Ae. aegypti plotted against abundance of Ae. albopictus in the preceding month (A) and vice versa (B). Table S1. Previous studies on Ae. aegypti abundance and urbanization. Table S2. Previous studies on Ae. albopictus abundance and urbanization. Table S3: Sequence of primers used for viral detection. Table S4. Model comparison of different radii for urbanization index predicting the number of larval habitat containers. Table S5. Model comparison of different radii for urbanization index predicting the number of larval habitat containers with mosquito larvae. Table S6. Model comparison of different radii for urbanization index predicting the number of human hosts. Table S7. Model comparison of different radii for urbanization index predicting the number of animal hosts. Table S8. Model comparison of different radii for urbanization index predicting the abundance of Ae. aegypti mosquitoes. Table S9. Analysis of Ae. aegypti abundance in six cities with urbanization index (UI), with temperature, humidity, and the sampling month’s rain as predictors. Table S10. Analysis of Ae. aegypti abundance in five cities (all but Yaounde) with structures within 100 m, city, temperature, humidity, and sampling month’s rain as predictors. Table S11. Analysis of Ae. aegypti abundance with number of humans, temperature, humidity, and monthly rainfall as predictors. Table S12. Model comparison of different radii for urbanization index predicting the abundance of Ae. albopictus mosquitoes. Table S13. Analysis of Ae. albopictus abundance in the three cities where this species was foun [file 13071_2025_6764_MOESM1_ESM.docx]

**Supplemental information**

**The effects of urbanization on *Aedes aegypti* and *Ae. albopictus* abundance across a broad latitudinal gradient in Central Africa**Matthew Montgomery^1,2^, James F. Harwood^1^, Aurelie P. Yougang^3^, Theodel A. Wilson-Bahun^3^, Armel N. Tedjou^3^, Christophe Rostand Keumeni^3^, Charles S. Wondji^3^, Basile Kamgang^3,^ A. Marm Kilpatrick^2^

**Supplemental Methods text**

**Pathogen detection.** We retrotranscribed extracted RNAs into cDNAs using the High-Capacity cDNA Reverse Transcription kit, 1000 reactions (Applied Biosystems, Foster city, California, USA). We prepared a mixture of 50 µL final volume, including 25 µL of RNA sample, 5 µL of 10X reverse transcription buffer, 2µL of 100 mM dNTPs, 5µL of 10X hexa random primers 10.5µL, 2.5 µL of reverse transcriptase, and 10.5 µL of RNAse-free H_2_O. We incubated the samples for 10 min at 25°C and 1 h at 37°C.

We performed quantitative real-time PCR using TaqMan Universal PCR Master Mix reagents (Applied Biosystems, Foster city, California, USA) to amplify UTR genes for Zika (Conceição *et al.*, 2010; Grard *et al.*, 2014) and dengue viruses (Leparc-Goffart *et al.*, 2009), and the E1 envelope protein for chikungunya virus (Ngoagouni *et al.*, 2017) (Table S3). Each PCR was performed in a 25 µL reaction mixture containing 12.5µL of 2x PCR TaqMan Universal PCR Master Mix, 1µL of each primer, 1 µL of each TaqMan probe (Applied Biosystems, Foster city, California, USA), 4.5 µL of RNAse-free H_2_O, and 5 µL template of cDNA. The amplification program consisted of a pre-activation heat step of 2 min at 50°C followed by 10 min at 95°C, and the amplification and fluorescence quantification steps of 45 cycles of 15 sec at 95°C and 1 min at 60°C. All assays were done on the Stratagene MX3005P qPCR machine (Agilent Technologies, Santa Clara, California, USA).

**Supplemental Tables and Figures**

**Table S1. Previous studies on *Ae. aegypti* abundance and urbanization.**

| **Study** | **Location** | **Urbanization Metric** | **Urbanization Correlation w/*Ae. aegypti* Abundance** |
| --- | --- | --- | --- |
| **Braks et al. 2003** | Rio de Janeiro & Nova Iguacu, Brazil; Boca Raton & West Palm Beach, FL, USA | Qualitative (Urban, suburban, rural) | Positive |
| **Carbajo et al. 2006** | Buenos Aires, Argentina | Quantitative GIS Land Cover Analysis (100m and 300m) | Positive |
| **Rey et al. 2006** | Manatee, Miami-Dade, & Palm Beach Co., FL, USA | Quantitative GIS Land Cover Analysis (100m) | Positive |
| **Tsuda et al. 2006** | Chiangmai Province, Thailand | Qualitative (Urban, Transition, Rural) | Positive |
| **Honorio et al. 2009** | Rio de Janeiro, Brazil | Qualitative (Urban, Low Vegetation, Medium Vegetation, High Vegetation) | Positive |
| **Higa et al. 2010** | Vietnam | Qualitative (Urban, Transition, Rural) | Mixed (regional variation) |
| **Fatima et al. 2016** | Pakistan | Quantitative GIS Land Cover Analysis (30m) | Positive |
| **Zahouli et al. 2016** | Cote D’Ivoire | Qualitative (Urban, Suburban, Rural) | Positive |
| **Ndenga et al. 2017** | Kenya | Qualitative (Urban, rural) | Positive |
| **Overgaard et al. 2017** | Colombia | Qualitative (Urban, rural) | Positive |
| **Dalpadado et al. 2018** | Gampaha District, Sri Lanka | Qualitative (Urban, Suburban, Rural) | Positive |
| **Estallo et al. 2018** | Cordoba, Argentina | Quantitative GIS Land Cover Analysis (10m) | Positive |
| **Talaga et al. 2020** | Kourou, French Guiana | Quantitative GIS Land Cover Analysis (70m) | Moderately Urbanized site had highest density |

**Table S2. Previous studies on *Ae. albopictus* abundance and urbanization. Study number indicates study location and result in Figure S4.**

| # | **Study** | **Location** | **Urbanization Metric** | **Urbanization Correlation w/*Ae. albopictus* Abundance** |
| --- | --- | --- | --- | --- |
| 1 | **Hornby et al. 1994** | Lee County, FL, USA | Qualitative (Urban, Suburban) | Negative |
| 2 | **Barker et al. 2003** | Virginia, USA | Qualitative (Forest, Yard Bordering Forest, Yard) | Positive (Unforested sites had highest abundance) |
| 3 | **Braks et al. 2003** | Rio de Janeiro & Nova Iguacu, Brazil; Boca Raton & West Palm Beach, FL, USA | Qualitative (Urban, suburban, rural) | Negative |
| 4 | **Rey et al. 2006** | Manatee, Miami-Dade, & Palm Beach Co., FL, USA | Quantitative GIS Land Cover Analysis (100m) | Negative |
| 5 | **Tsuda et al. 2006** | Chiangmai Province, Thailand | Qualitative (Urban, Transition, Rural) | Negative |
| 6 | **Honorio et al. 2009** | Rio de Janeiro, Brazil | Qualitative (Urban, Low Vegetation, Medium Vegetation, High Vegetation) | Negative |
| 7 | **Higa et al. 2010** | Vietnam | Qualitative (Urban, Transition, Rural) | None |
| 8 | **Bagny et al. 2012** | Mayotte, France | Quantitative GIS Land Cover Analysis (25m) | Positive |
| 9 | **Li et al. 2014** | Guangzho, China | Qualitative (Urban, suburban, rural) | Positive |
| 10 | **Samson et al. 2015** | Cap-Haitien, Haiti | Quantitative GIS Land Cover Analysis (6.5m) | Positive |
| 11 | **Baldacchino et al. 2017** | Belluno & Trento, Italy | Quantitative GIS Land Cover Analysis (250m) | Positive |
| 12 | **Dalpadado et al. 2018** | Gampaha District, Sri Lanka | Qualitative (Urban, Suburban, Rural) | Negative |
| 13 | **McClure et al. 2018** | Big Island, Hawaii, USA | Quantitative GIS Land Cover Analysis | Positive |
| 14 | **Arduino et al. 2020** | Sao Paulo, Brazil | Qualitative (Urban, Forest, Grass-Shrubs) | Positive |
| 15 | **Westby et al. 2021** | St. Louis, Missouri, USA | Qualitative (Urban, Suburban, Rural) | Positive (Urban & Suburban equal, but both higher than rural) |

**Table S3. Sequence of primers used for viral detection.**

| **Primers** | **Sequence (5’-3’)** | **References** |
| --- | --- | --- |
| ZIKV-forward | nt9271-AARTACACATACCARAACAAAgTggT9297 | (Lanciotti *et al.*, 2008) |
| ZIKV-reverse | nt9352-TCCRCTCCCYCTYTggTCTTg-9373 |  |
| ZIKV-probe | nt9304-FAM-CTYAgACCAgCTgAAR-BBQ-9320 |  |
| CHIKV-forward | AAGCTYCGCGTCCTTTACCAAG | (Pastorino *et al.*, 2005) |
| CHIKV-reverse | CCAAATTGTCCYGGTCTTCCT |  |
| CHIKV-probe | FAM-CCAATGTCYTCMGCCTGGACACCTTT-TAMRA |  |
| DENV-forward | AGGACYAGAGGTTAGAGGAGA | (Leparc-Goffart *et al.*, 2009) |
| DENV-reverse | CGYTCTGTTGCCTGGAWTGAT |  |
| DENV-probe | FAM-ACAGCATATTGACGCTGGGARAGACC-TAMRA |  |

**Table S4. Model comparison of different radii for urbanization index predicting the number of larval habitat containers. All models included urbanization at a given radius and site as a random effect.**

| Radius | AIC | Delta_AIC | AICWt |
| --- | --- | --- | --- |
| UICTest2KM | 782.03 | 0 | 0.38 |
| UICTest500M | 782.74 | 0.71 | 0.26 |
| UICTest1KM | 783.7 | 1.67 | 0.16 |
| UICTest200M | 783.99 | 1.96 | 0.14 |
| UICTest100M | 785.98 | 3.95 | 0.05 |

**Table S5. Model comparison of different radii for urbanization index predicting the number of larval habitat containers with mosquito larvae. All models included urbanization at a given radius and site as a random effect.**

| Radius | AIC | Delta_AIC | AICWt |
| --- | --- | --- | --- |
| UIPCTest2KM | 646 | 0 | 0.5 |
| UIPCTest1KM | 647.87 | 1.87 | 0.2 |
| UIPCTest500M | 648.19 | 2.19 | 0.17 |
| UIPCTest200M | 649.28 | 3.28 | 0.1 |
| UIPCTest100M | 651.6 | 5.59 | 0.03 |

**Table S6. Model comparison of different radii for urbanization index predicting the number of human hosts. All models included urbanization at a given radius and site as a random effect.**

| Radius | AIC | Delta_AIC | AICWt |
| --- | --- | --- | --- |
| 1KM | 302.03 | 0 | 0.47 |
| 500M | 303.32 | 1.28 | 0.25 |
| 2KM | 303.67 | 1.63 | 0.21 |
| 200M | 306.39 | 4.36 | 0.05 |
| 100M | 307.59 | 5.55 | 0.03 |

**Table S7. Model comparison of different radii for urbanization index predicting the number of animal hosts. All models included urbanization at a given radius and site as a random effect.**

| Radius | AIC | Delta_AIC | AICWt |
| --- | --- | --- | --- |
| 200M | 134.19 | 0 | 0.68 |
| 500M | 137.15 | 2.97 | 0.15 |
| 100M | 137.56 | 3.38 | 0.13 |
| 1KM | 140.21 | 6.03 | 0.03 |
| 2KM | 142.33 | 8.14 | 0.01 |

**Table S8. Model comparison of different radii for urbanization index predicting the abundance of *Ae. aegypti* mosquitoes. All models included urbanization at a given radius, city, temperature, humidity, the current month’s rainfall, and site as a random effect.**

| Radius | AIC | Δ_AIC | AIC Weight |
| --- | --- | --- | --- |
| 1KM | 904.67 | 0 | 0.42 |
| 2KM | 905.26 | 0.59 | 0.31 |
| 500m | 906.09 | 1.42 | 0.2 |
| 100m | 909.57 | 4.9 | 0.04 |
| 200m | 909.8 | 5.12 | 0.03 |

**Table S9. Analysis of *Ae. aegypti* abundance in six cities with urbanization index (UI), with temperature, humidity, and the sampling month’s rain as predictors in a generalized linear mixed effects model with negative binomial distribution, a log link, and site as a random effect (estimated variance: 0.64)*.* The city of Douala was the reference level (variation among Cities was highly significant; City: χ^2^ = 33.0, df = 5, P = 3.7 × 10^-6^).**

| Predictor | Coef. | SE | Z | P-value |
| --- | --- | --- | --- | --- |
| Intercept | 1.31 | 2.06 | 0.64 | 0.53 |
| UI (1KM) | 0.017 | 0.0050 | 3.33 | 0.00088 |
| City: Garoua | -0.77 | 0.54 | -1.41 | 0.16 |
| City: Kribi | -1.15 | 0.53 | -2.16 | 0.031 |
| City: Maroua | -1.02 | 0.56 | -1.81 | 0.070 |
| City: Ngaoundere | -1.33 | 0.59 | -2.26 | 0.024 |
| City: Yaounde | -2.82 | 0.51 | -5.56 | 2.69x10^-8^ |
| Temperature | -0.032 | 0.052 | -0.61 | 0.54 |
| Humidity | 0.0019 | 0.009 | 0.217 | 0.83 |
| Rain (mm) | 0.0019 | 0.0008 | 2.38 | 0.017 |

**Table S10. Analysis of *Ae. aegypti* abundance in five cities (all but Yaounde) with structures within 100m, city, temperature, humidity, and sampling month’s rain as predictors in a generalized linear mixed effects model with negative binomial distribution, a log link, and site as a random effect (estimated variance: 2.2 × 10^-9^; a model without site was nearly identical)*.* The city of Douala was the reference level (variation among Cities was significant; City: χ = 187.8, df = 4, P = 0.0013).**

| Predictor | Coef. | SE | Z | P-value |
| --- | --- | --- | --- | --- |
| (Intercept) | 0.80 | 2.06 | 0.39 | 0.70 |
| Structures (100m) | 0.0040 | 0.0023 | 1.71 | 0.088 |
| City: Garoua | -1.30 | 0.48 | -2.71 | 0.0068 |
| City: Kribi | -1.58 | 0.42 | -3.77 | 0.00017 |
| City: Maroua | -1.26 | 0.41 | -3.05 | 0.0023 |
| City: Ngaoundere | -1.55 | 0.52 | -2.98 | 0.0029 |
| Temperature | 0.043 | 0.054 | 0.80 | 0.42 |
| Humidity | -0.00089 | 0.0081 | -0.11 | 0.91 |
| Rain (mm) | 0.0018 | 0.0011 | 1.60 | 0.11 |

**Table S11.** **Analysis of *Ae. aegypti* abundance with number of humans, temperature, humidity, and monthly rainfall as predictors in the three cities where human surveys were done using a generalized linear mixed effects model with negative binomial distribution, a log link, and site as a random effect (estimated variance: 0.83)*.* The city of Douala was the reference level (variation among Cities was significant; City: χ = 22.6, df = 2, P = 0.000012).**

| Predictor | Coef. | SE | Z | P-value |
| --- | --- | --- | --- | --- |
| (Intercept) | 4.25 | 3.98 | 1.07 | 0.29 |
| Humans | 0.022 | 0.0072 | 3.04 | 0.0024 |
| City: Kribi | -1.02 | 0.65 | -1.57 | 0.12 |
| City: Yaounde | -3.06 | 0.66 | -4.63 | 3.68E-06 |
| Temperature | -0.12 | 0.099 | -1.22 | 0.22 |
| Humidity | -0.0076 | 0.018 | -0.43 | 0.67 |
| Rain (mm) | 0.0034 | 0.0011 | 3.09 | 0.0020 |

**Table S12. Model comparison of different radii for urbanization index predicting the abundance of *Ae. albopictus* mosquitoes. All models included urbanization at a given radius, city, temperature, humidity, the previous month’s rainfall, and site as a random effect.**

| Radius | AIC | Δ_AIC | AIC Weight |
| --- | --- | --- | --- |
| 100m | 1523.73 | 0 | 0.55 |
| 2Km | 1524.64 | 0.91 | 0.35 |
| 200m | 1528.75 | 5.03 | 0.04 |
| 1Km | 1529.24 | 5.51 | 0.04 |
| 500m | 1530.91 | 7.18 | 0.02 |

**Table S13. Analysis of *Ae. albopictus* abundance in the three cities where this species was found, with urbanization index (UI) interacting with city, and temperature, humidity, and prior month’s rain as predictors in a generalized mixed effects linear model with a negative binomial distribution a log link, and site as a random effect (estimated variance: 2.6 x 10^-9^; a model without a random effect was nearly identical)*.* The city of Yaounde was the reference level. There was significant variation in *Ae. albopictus* abundance among cities (χ = 6.24; df = 2; P = 0.044) and in the slope of urbanization among cites (χ = 14.3; df = 2; P = 0.00078); the slope of UI was not significant in either Douala (slope -0.0047; SE = 0.0078; P = 0.54) or Kribi (slope 0.0025; SE = 0.0042; P = 0.55).**

| Predictor | Coef. | SE | Z | P-value |
| --- | --- | --- | --- | --- |
| (Intercept) | -2.56 | 1.64 | -1.56 | 0.12 |
| UI (100m) | 0.017 | 0.0023 | 7.28 | 3.31E-13 |
| City: Douala | 0.64 | 0.64 | 1.00 | 0.32 |
| City: Kribi | 0.58 | 0.34 | 1.70 | 0.088 |
| Temperature | 0.090 | 0.043 | 2.10 | 0.04 |
| Humidity | 0.020 | 0.0079 | 2.56 | 0.01 |
| Prior Month Rain (mm) | 0.0017 | 0.00040 | 4.17 | 0.00003 |
| UI (100m):Douala | -0.022 | 0.0081 | -2.67 | 0.0075 |
| UI (100m):Kribi | -0.014 | 0.0048 | -3.03 | 0.0025 |

**Table S14. Analysis of *Ae. albopictus* abundance in the two cities where this species was found, and larval habitat studies were performed (Douala and Kribi), with larval containers, city, temperature, humidity, and prior month’s rain as predictors in a generalized mixed effects linear model with a negative binomial distribution a log link, and site as a random effect (estimated variance: 0.054)*.* The city of Douala was the reference level.**

| Predictor | Coef. | SE | Z | P-value |
| --- | --- | --- | --- | --- |
| (Intercept) | -4.41 | 8.18 | -0.54 | 0.59 |
| Containers | 0.013 | 0.0043 | 3.11 | 0.0019 |
| City: Kribi | 12.92 | 7.41 | 1.74 | 0.081 |
| Temperature | 0.22 | 0.14 | 1.54 | 0.12 |
| Humidity | 0.059 | 0.022 | 2.66 | 0.0078 |
| Prior Month Rain (mm) | -0.066 | 0.045 | -1.47 | 0.14 |

**Table S15. Analysis of *Ae. albopictus* abundance in the two cities where this species was found, and larval habitat studies were performed (Douala and Kribi), with larval containers with mosquito larvae (pos. containers), city, temperature, humidity, and prior month’s rain as predictors in a generalized mixed effects linear model with a negative binomial distribution a log link, and site as a random effect (estimated variance: 0.052)*.* The city of Douala was the reference level.**

| Predictor | Coef. | SE | Z | P-value |
| --- | --- | --- | --- | --- |
| (Intercept) | -2.96 | 8.52 | -0.35 | 0.73 |
| Pos. containers | 0.027 | 0.010 | 2.66 | 0.0079 |
| City: Kribi | 12.99 | 7.97 | 1.63 | 0.10 |
| Temperature | 0.17 | 0.13 | 1.29 | 0.20 |
| Humidity | 0.052 | 0.022 | 2.43 | 0.015 |
| Prior Month Rain (mm) | -0.065 | 0.049 | -1.34 | 0.18 |

**Table S16. Analysis of *Ae. albopictus* abundance in the two cities where this species was found, and larval habitat studies were performed (Douala and Kribi), with larval containers with *Ae. albopictus* mosquito larvae (Alb. larvae), city, temperature, humidity, and prior month’s rain as predictors in a generalized mixed effects linear model with a negative binomial distribution a log link, and site as a random effect (estimated variance: 0.033)*.* The city of Douala was the reference level.**

| Predictor | Coef. | SE | Z | P-value |
| --- | --- | --- | --- | --- |
| (Intercept) | -0.80 | 7.86 | -0.10 | 0.92 |
| Alb. larvae | 0.0016 | 0.00051 | 3.07 | 0.0022 |
| City: Kribi | 13.39 | 7.69 | 1.74 | 0.082 |
| Temperature | 0.17 | 0.13 | 1.24 | 0.22 |
| Humidity | 0.041 | 0.021 | 1.95 | 0.052 |
| Prior Month Rain (mm) | -0.071 | 0.046 | -1.54 | 0.12 |

**Table S17. Analysis of *Ae. albopictus* abundance in the two cities where this species was found, and larval habitat studies were performed (Douala and Kribi), with the fraction of larval containers containing mosquito larvae (Cont. index), city, temperature, humidity, and prior month’s rain as predictors in a generalized mixed effects linear model with a negative binomial distribution a log link, and site as a random effect (estimated variance: 1.3 x 10^-8^)*.* The city of Douala was the reference level.**

| Predictor | Coef. | SE | Z | P-value |
| --- | --- | --- | --- | --- |
| (Intercept) | 4.99 | 7.53 | 0.66 | 0.51 |
| Cont. index | -1.58 | 0.75 | -2.10 | 0.036 |
| City: Kribi | 17.66 | 7.25 | 2.44 | 0.015 |
| Temperature | 0.13 | 0.14 | 0.93 | 0.35 |
| Humidity | 0.042 | 0.020 | 2.16 | 0.031 |
| Prior Month Rain (mm) | -0.10 | 0.043 | -2.33 | 0.020 |

**Table S18. Analysis of *Ae. albopictus* abundance in the two cities where this species was found, and larval habitat studies were performed (Douala and Kribi), with Structures within 100m, city, temperature, humidity, and prior month’s rain as predictors in a generalized mixed effects linear model with a negative binomial distribution a log link, and site as a random effect (estimated variance: 0.16)*.* The city of Douala was the reference level.**

| Predictor | Coef. | SE | Z | P-value |
| --- | --- | --- | --- | --- |
| (Intercept) | 6.82 | 7.51 | 0.91 | 0.36 |
| Structures_100M | -0.010 | 0.0061 | -1.72 | 0.086 |
| City: Kribi | 16.71 | 7.31 | 2.29 | 0.022 |
| Temperature | 0.086 | 0.13 | 0.67 | 0.50 |
| Humidity | 0.026 | 0.021 | 1.21 | 0.23 |
| Prior Month Rain (mm) | -0.097 | 0.044 | -2.23 | 0.026 |

**Table S19. Analysis of *Ae. albopictus* abundance in the three cities where this species was found, with humans, city, temperature, humidity, and prior month’s rain as predictors in a generalized mixed effects linear model with a negative binomial distribution a log link, and site as a random effect (estimated variance: 0.087)*.* The city of Douala was the reference level.**

| Predictor | Coef. | SE | Z | P-value |
| --- | --- | --- | --- | --- |
| (Intercept) | -3.66 | 2.04 | -1.80 | 0.073 |
| Humans | 0.011 | 0.0030 | 3.56 | 0.00038 |
| City: Kribi | 1.20 | 0.55 | 2.19 | 0.028 |
| City: Yaounde | 1.43 | 0.54 | 2.64 | 0.0082 |
| Temperature | 0.11 | 0.047 | 2.24 | 0.025 |
| Humidity | 0.019 | 0.0083 | 2.26 | 0.024 |
| Prior Month Rain (mm) | 0.0018 | 0.00044 | 4.08 | 4.49E-05 |

**Table S20. Compiled Minimum Infection Rates (MIR) for studies which detected Zika virus in field caught specimens of *Ae. aegypti* and *Ae. albopictus*.**

| **Study** | **Country** | **Species** | **Zika+ Pools** | **Total Pools** | **Mosq. Tested** | **Max Pool Size** | **MIR** |
| --- | --- | --- | --- | --- | --- | --- | --- |
| **Marchette 1969** | Malaysia | *Ae. aegypti* | 1 | 58 | 1277 | 80 | 0.8 |
| **Grard 2014** | Gabon | *Ae. albopictus* | 2 | 91 | 2130 | 25 | 0.9 |
| **Ferreira-de-Brito 2016** | Brazil | *Ae. aegypti* | 3 | 198 | 550 | 10 | 5.5 |
| **Guerbois 2016** | Mexico | *Ae. aegypti* | 15 | 55 | 279 | NA | 53.8 |
| **Ho 2017** | Singapore | *Ae. aegypti, Ae. albopictus*** | 9 | 517 | 1375 | 5 | 6.5 |
| **Cevallos 2018** | Ecuador | *Ae. aegypti* | 2 | 14 | 193 | 10 | 10.4 |
| **Correa-Morales 2019** | Mexico | *Ae. aegypti* | 260 | 3120 | 14,145 | NA | 18.4 |
| **Correa-Morales 2019** | Mexico | *Ae. albopictus* | 7 | 52 | 78 | NA | 89.7 |
| **Singh 2019** | India | *Ae. aegypti* | 3 | 55 | 203 | 10 | 14.8 |
| **Ali 2020** | Malaysia | *Ae. albopictus* | 6 | NA | 186 | 25 | 32.3 |
| **Calle-Tobon 2020** | Colombia | *Ae. aegypti* | 98 | NA | 6585 | 10 | 14.9 |
| **Campos 2020** | Cape Verde | *Ae. aegypti* | 2 | 816 | 816 | 1 | 2.5 |
| **Kosoltanapawit 2020** | Thailand | *Ae. aegypti* | 2 | 130 | 130 | 1 | 15.4 |
| **Phumee 2020*** | Thailand | *Ae. aegypti* | NA | NA | NA | 10 | 22.4 |
| **Parra 2022** | Sao Paulo, Brazil | *Ae. aegypti* | 55 | 607 | 1026 | 10 | 53.6 |
| **Parra 2022** | Sao Paulo, Brazil | *Ae. albopictus* | 1 | 11 | 12 | 10 | 83.3 |
| **Akyea-Bobi 2023** | Ghana | *Ae. aegypti* | 0 | 119 | 1493 | 20 | 0 |
| **This Study** | Cameroon | *Ae. albopictus* | 0 | 289 | 7771 | 30 | 0 |
| **This Study** | Cameroon | *Ae. aegypti* | 0 | 113 | 1660 | 30 | 0 |

- Study provided an MIR without number of specimens or pools tested.

* Study data did not allow for differentiation of *Ae. aegypti* and *Ae. albopictus* so results were combined.


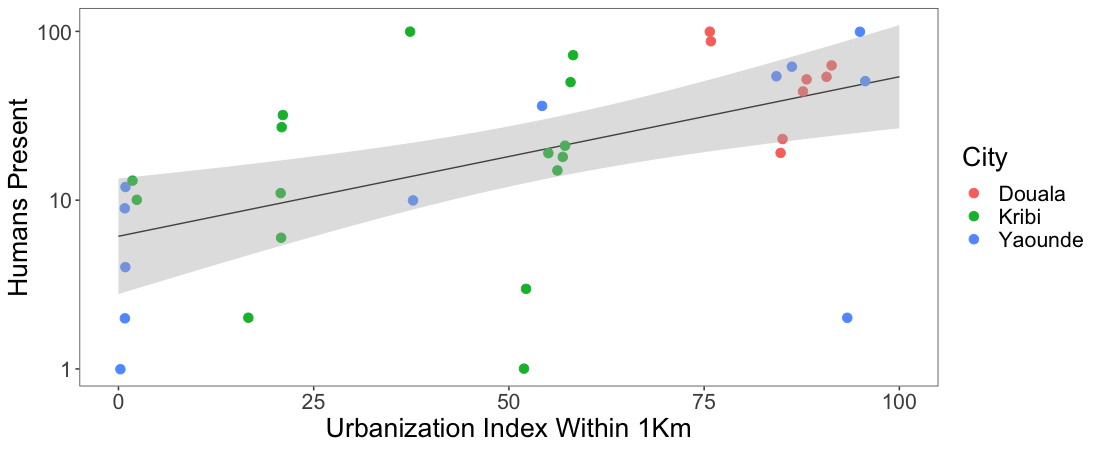


**Figure S1. Humans observed at collection sites in Douala, Kribi, and Yaounde. The line and ribbon show the fitted model and 95% CI, respectively.**


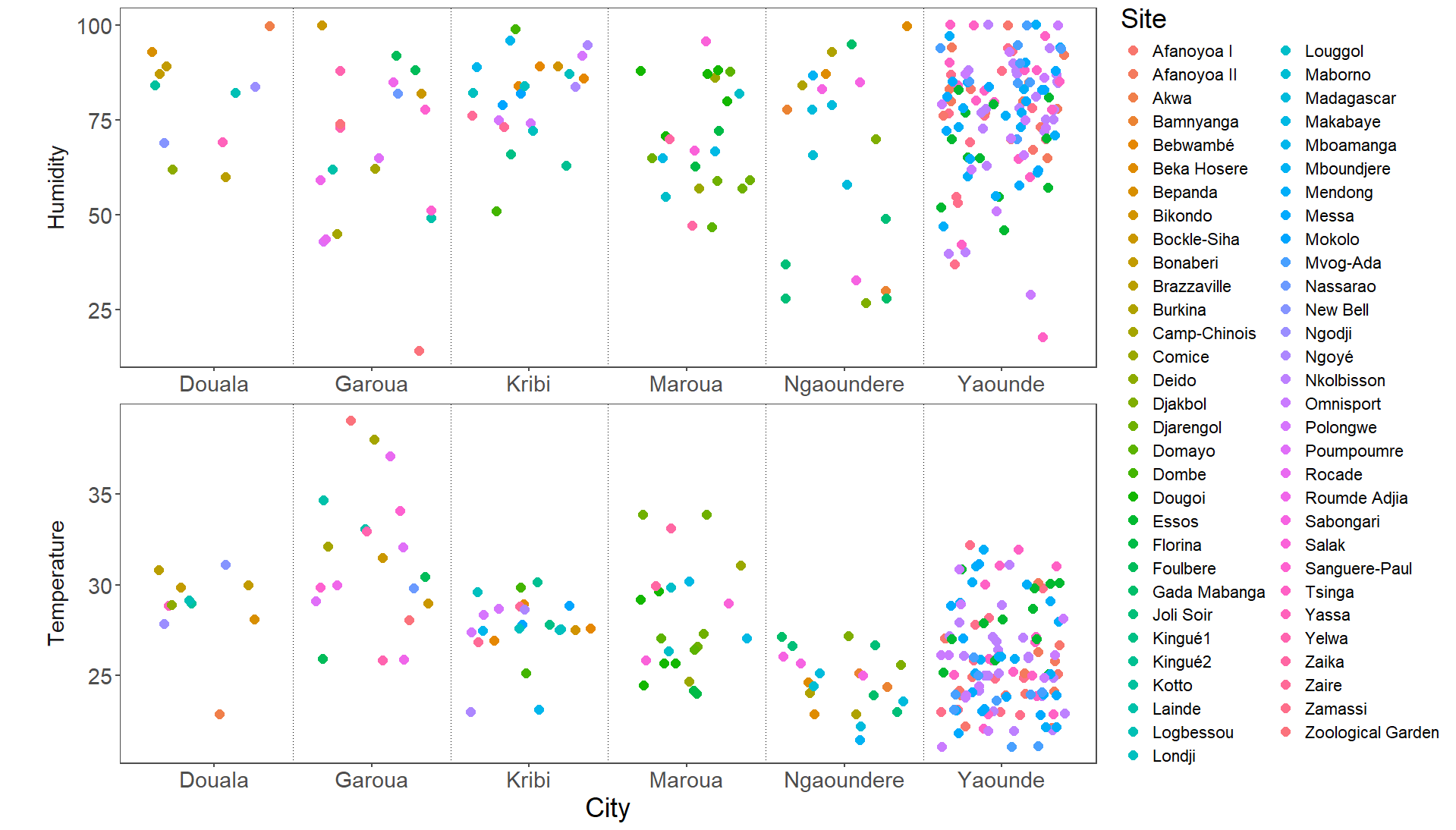


**Figure S2. Relative humidity (in percent) and temperature (in degrees Celsius) measurements from each of the six cities. Each point is a measurement at a site. A small amount of jitter, primarily in the horizontal direction, has been added to each point to facilitate visualization.**


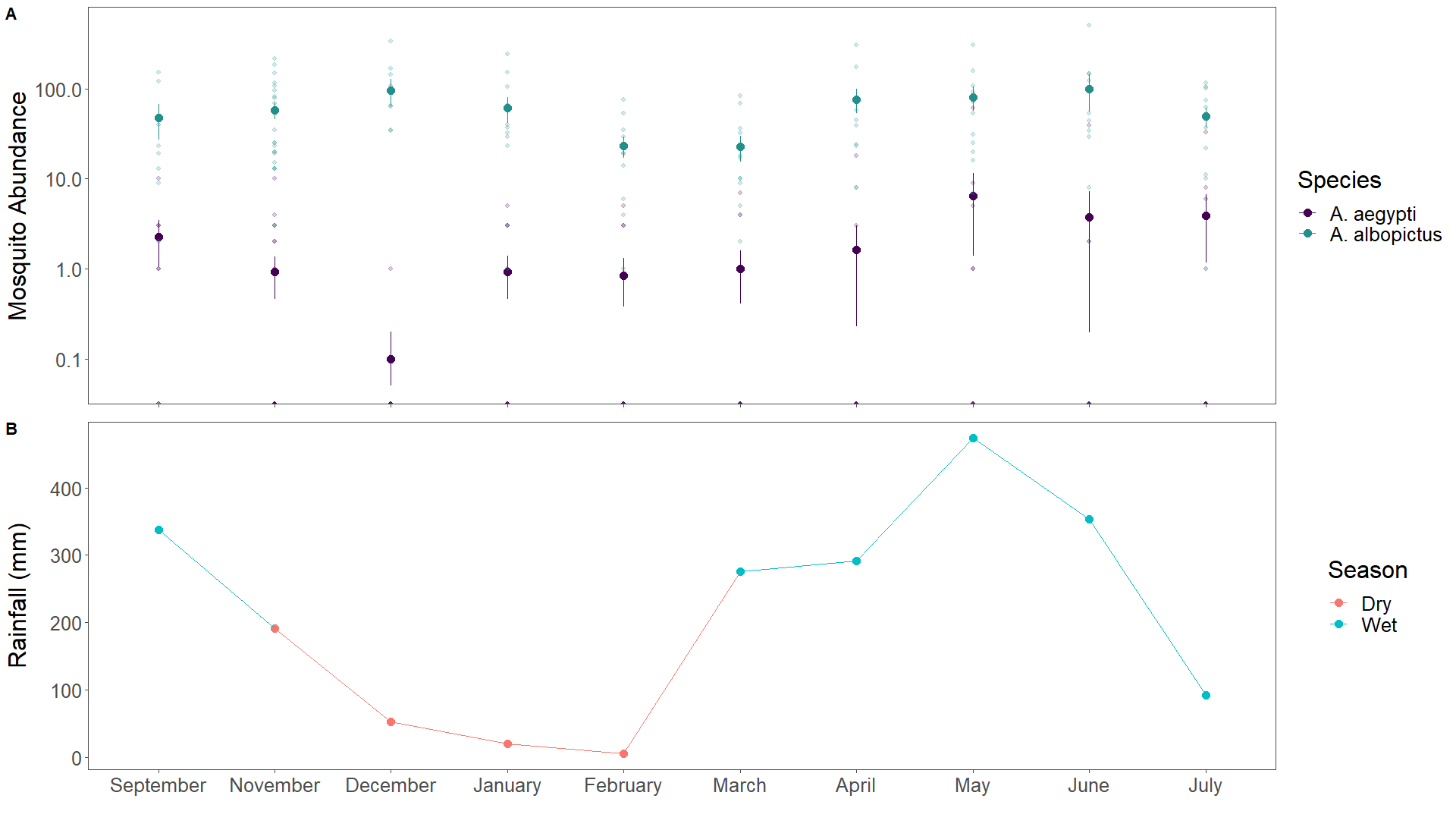


**Figure S3. Monthly rainfall and mosquito abundance (mosquitoes/3 hr) for ten sites in Yaounde. Dark circles represent the mean catch rate per hour across all sites (+/- 1SE).**


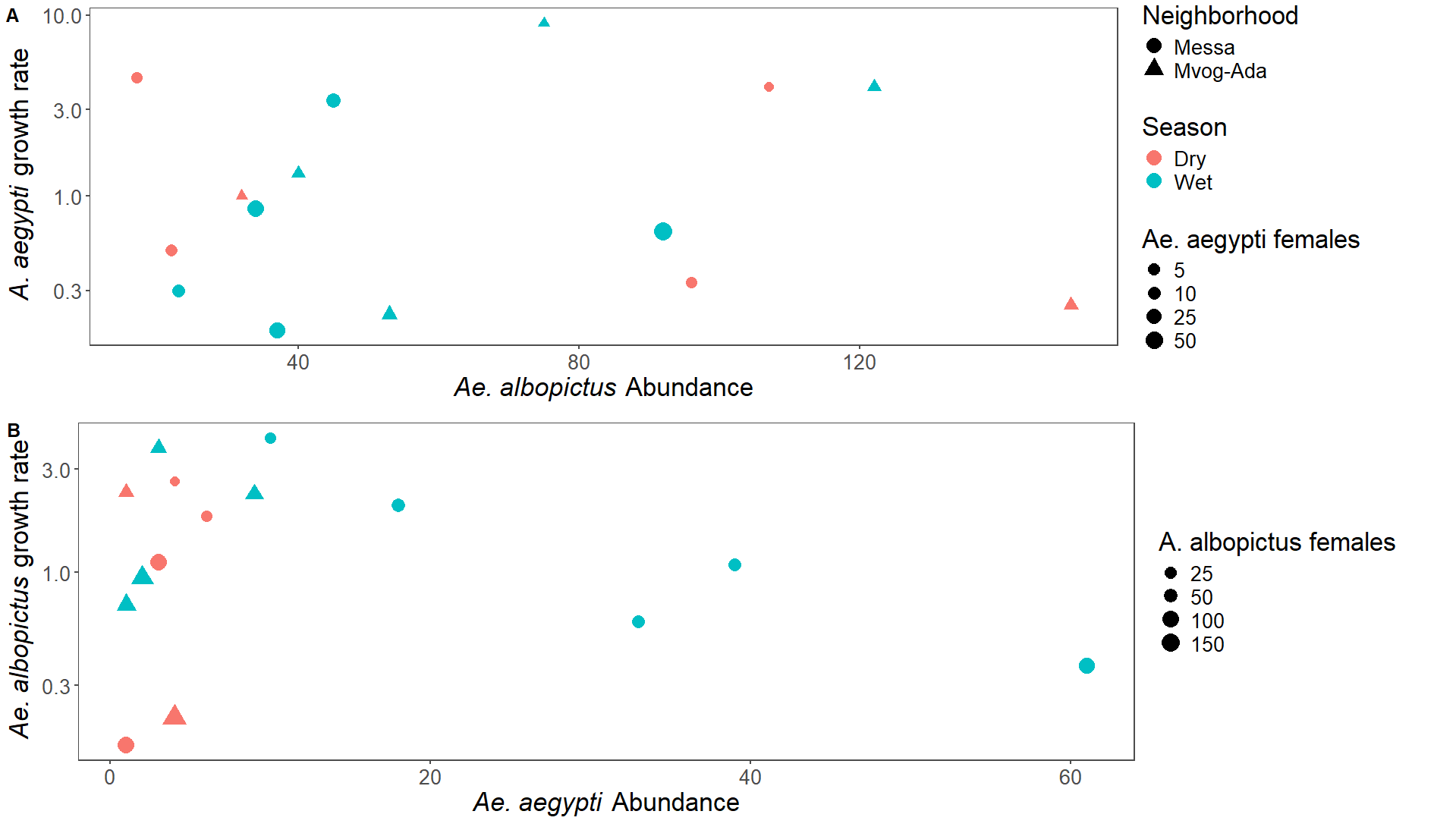


**Figure S4. Monthly population growth rate of *Ae. aegypti* plotted against abundance of *Ae. albopictus* in the preceding month (A) and vice versa (B), plotted on a log scale. There was no detectable effect of *Ae. albopictus* on *Ae. aegypti* population growth rates (A) (Log(Ae. aegypti pop. growth rate) = 0.082 - 0.0089*Ae. aegypti (SE = 0.0087) + 0.00042*Ae. albopictus (SE = 0.0037); P = 0.91 and P = 0.33 respectively). B) There was weak evidence of competition with a negative correlation between *Ae. albopictus* population growth rate and *Ae. aegypti* abundance in the previous month, when *Ae. albopictus* in the previous month was included in the model (Log(*Ae. albopictus* pop. growth rate) = 0.67 -0.0083**Ae. albopictus* (SE = 0.0017) -0.0074**Ae. aegypti* (SE = 0.0041); P = 0.00046 and P = 0.095, respectively).**


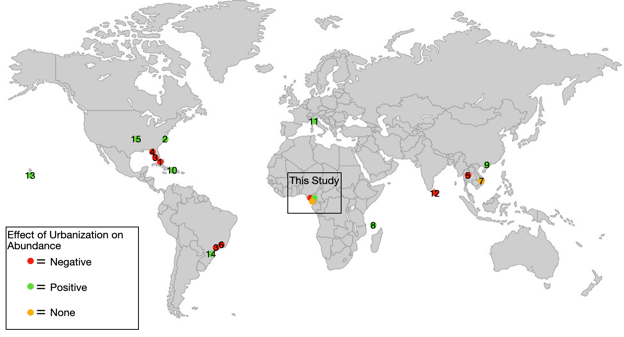


**Figure S5. Locations of studies of *Ae. albopictus* abundance in response to urbanization. Numbers correspond to study referenced in Table S2.**
